# Maternal dietary intake of fish and PUFAs and child neurodevelopment at 6 months and 1 year of age: a nationwide birth cohort—the Japan Environment and Children's Study (JECS)

**DOI:** 10.1093/ajcn/nqaa190

**Published:** 2020-08-07

**Authors:** Kei Hamazaki, Kenta Matsumura, Akiko Tsuchida, Haruka Kasamatsu, Tomomi Tanaka, Mika Ito, Hidekuni Inadera, Japan Environment and Children's Study Group, Michihiro Kamijima, Shin Yamazaki, Yukihiro Ohya, Reiko Kishi, Nobuo Yaegashi, Koichi Hashimoto, Chisato Mori, Shuichi Ito, Zentaro Yamagata, Hidekuni Inadera, Takeo Nakayama, Hiroyasu Iso, Masayuki Shima, Youichi Kurozawa, Narufumi Suganuma, Koichi Kusuhara, Takahiko Katoh

**Affiliations:** Department of Public Health, Faculty of Medicine, University of Toyama, Toyama, Japan; Toyama Regional Center for JECS, University of Toyama, Toyama, Japan; Toyama Regional Center for JECS, University of Toyama, Toyama, Japan; Department of Public Health, Faculty of Medicine, University of Toyama, Toyama, Japan; Toyama Regional Center for JECS, University of Toyama, Toyama, Japan; Toyama Regional Center for JECS, University of Toyama, Toyama, Japan; Toyama Regional Center for JECS, University of Toyama, Toyama, Japan; Department of Pediatrics, Faculty of Medicine, University of Toyama, Toyama, Japan; Department of Obstetrics and Gynecology, Faculty of Medicine, University of Toyama, Toyama, Japan; Department of Public Health, Faculty of Medicine, University of Toyama, Toyama, Japan; Toyama Regional Center for JECS, University of Toyama, Toyama, Japan; Nagoya City University, Nagoya, Japan; National Institute for Environmental Studies, Tsukuba, Japan; National Center for Child Health and Development, Tokyo, Japan; Hokkaido University, Sapporo, Japan; Tohoku University, Sendai, Japan; Fukushima Medical University, Fukushima, Japan; Chiba University, Chiba, Japan; Yokohama City University, Yokohama, Japan; University of Yamanashi, Chuo, Japan; University of Toyama, Toyama, Japan; Kyoto University, Kyoto, Japan; Osaka University, Suita, Japan; Hyogo College of Medicine, Nishinomiya, Japan; Tottori University, Yonago, Japan; Kochi University, Nankoku, Japan; University of Occupational and Environmental Health, Kitakyushu, Japan; Kumamoto University, Kumamoto, Japan

**Keywords:** pregnancy, fish, polyunsaturated fatty acids, neurodevelopment, infant

## Abstract

**Background:**

Although emerging evidence indicates a relation between maternal intake of fish and improved child neurodevelopment, the results are inconsistent.

**Objectives:**

This study investigated whether dietary consumption of fish during pregnancy is associated with offspring neurodevelopment at age 6 mo and 1 y. As exploratory research, we also examined the association between consumption of PUFAs and neurodevelopment at the same time points.

**Methods:**

After exclusion and multiple imputation from a dataset comprising 104,065 records from the Japan Environment and Children's Study, we evaluated 81,697 and 77,751 mother-child pairs at age 6 mo and 1 y, respectively.

**Results:**

Maternal fish intake during pregnancy was independently associated with reduced risk of delay in problem-solving at age 6 mo (lowest compared with highest quintile OR = 0.88; 95% CI: 0.79, 0.99; *P*-trend = 0.01) and in fine motor skills (highest quintile OR = 0.90; 95% CI: 0.81, 0.99; *P*-trend = 0.02) and problem-solving (fourth quintile OR = 0.89; 95% CI: 0.81, 0.98; and highest quintile OR = 0.90; 95% CI: 0.81, 0.99; *P*-trend = 0.005) at age 1 y. Dietary intake of total n–3 PUFAs was associated with reduced risk of delay in fine motor skills at 6 mo, and in fine motor skills and problem-solving at 1 y. Dietary intake of total n–6 PUFAs was associated with reduced risk of delay in communication and fine motor skills at 6 mo, and in gross motor skills, fine motor skills, and problem-solving at 1 y. In contrast, the dietary n–6/n–3 ratio was positively associated with increased risk of delay in problem-solving at 1 y.

**Conclusions:**

The results of this study suggest there might be beneficial effects of fish intake during pregnancy on some domains of child psychomotor development and this effect might be partially explained by PUFA intake from fish. Trial registration: UMIN000030786.

See corresponding editorial on page 1149.

## Introduction

Numerous observational studies have found that dietary intake of fish and/or n–3 PUFAs during pregnancy is beneficial for child psychomotor development (1–10). However, other studies have found no effects (11, 12). Fish is known also to be contaminated with methylmercury, dioxins, polychlorinated biphenyls, and several other persistent halogenated organic pollutants, and among these contaminants mercury has detrimental effects on fetal neurodevelopment (13). Greiner et al. (14) conducted a survey in the United States analyzing 310 health-related news stories on fish and found that for every 1 benefit message, 4 risk messages appeared. A systematic review revealed that the benefits of diets providing moderate amounts of fish during pregnancy outweigh potential detrimental effects on offspring neurodevelopment (15). In Japan, information disseminated by the Ministry of Health, Labour, and Welfare encourages pregnant women to consume seafood for nutritional benefits (e.g., high-quality protein, DHA, and EPA) but also to pay attention to some species that might contain high mercury concentrations (16).

As far as we know, there have been only 2 observational studies conducted in Japan on the effects of diet during pregnancy on offspring development (17, 18). Suzuki et al. (17) found that total seafood intake during pregnancy was not significantly associated with motor cluster score on the Neonatal Behavioral Assessment Scale for 3-d-old infants (*n* = 498; *P* > 0.1). Miyake et al. (18) examined the association between maternal fatty acid consumption during pregnancy and behavioral problems in 1199 Japanese children at age 5 y; although they did not evaluate the effects of fish consumption specifically, the results for DHA and EPA, which mainly come from consuming fish, showed no significant associations (18). There is no clear explanation for these null associations, but we speculate that the Japanese population is consuming enough fish to mask the difference in findings between the overseas and Japanese studies. In addition, because these 2 studies were restricted to certain geographic areas in Japan (17, 18), a new study that is representative of Japanese pregnant women is warranted.

We hypothesized that fish intake during pregnancy in young mothers might enhance the psychomotor development of their children. To test this hypothesis, we used data from the Japan Environment and Children's Study (JECS), a national study, to examine whether dietary fish intake during pregnancy was associated with the psychomotor development of offspring at age 6 mo and 1 y. As exploratory research, we also examined the association between consumption of PUFAs and psychomotor development at the same time points.

## Methods

### Study population

**FIGURE 1:**
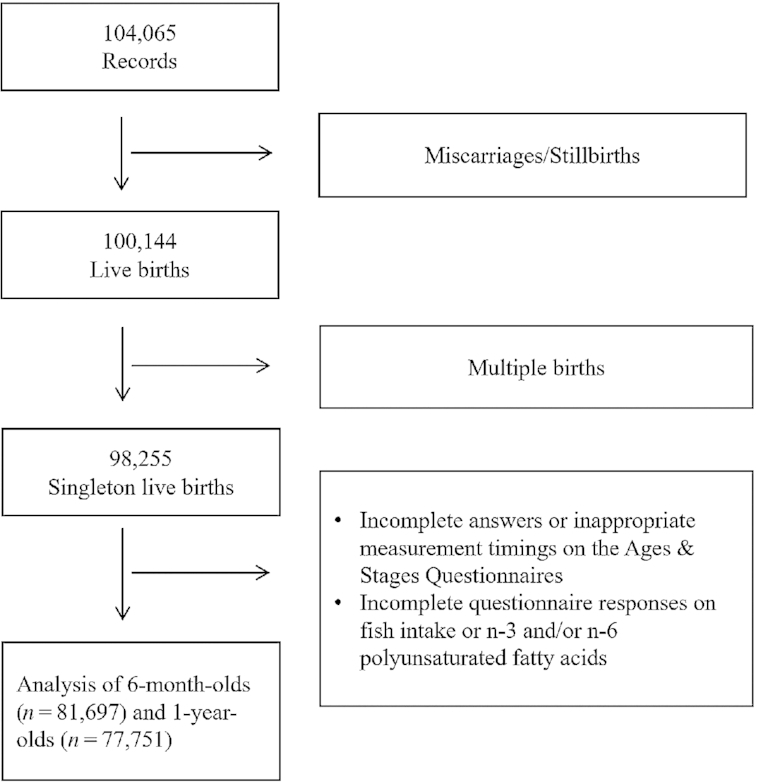
Flow diagram of the recruitment and exclusion process for the participants.

The JECS protocol is described in detail elsewhere (19, 20). Briefly, the aim of the JECS, a nationwide government-funded birth cohort study, is to evaluate the impact of certain environmental factors on child health and development. The pregnant participants in the JECS were enrolled from 15 Japanese regions from January 2011 to March 2014 (19, 20). The present study is based on the jecs-an-20,180,131 dataset, which was released in March 2018. The full dataset comprises 104,065 records, but we excluded 3921 and 1889 records because of miscarriages/stillbirths and multiple births, respectively (**Figure 1**). We also excluded 16,558 and 20,504 records because of incomplete answers and inappropriate measurement timings for the Ages & Stages Questionnaires (ASQ-3) and/or incomplete questionnaires on fish intake, n–3 PUFAs, and/or n–6 PUFAs. This left data from 81,697 and 77,751 mother-child pairs at age 6 mo and 1 y, respectively, for analysis.

The study protocol was approved by the Japanese Ministry of the Environment's Institutional Review Board on Epidemiological Studies and the ethics committees of all participating institutions. All pregnant participants provided written informed consent.

### Measurements of fish and PUFA intakes

In the JECS, dietary consumption of fish and total n–3 and n–6 PUFAs was determined by the FFQ, which is semiquantitative and has been validated for use in large-scale Japanese epidemiological studies (21). The FFQ was administered in mid–late pregnancy, and participants were asked to respond about their dietary intake in the period between learning of the pregnancy and the second/third trimester. Of the 171 food and beverage items, 21 concern fish or shellfish consumption. Participants answered how often they consumed each food type during the mid–late pregnancy (covering dietary intake after they learned of the pregnancy). The standard portion size for each food type was categorized as small (50% smaller than standard), medium (same as standard), or large (50% larger than standard). For each of the 21 items of fish or shellfish, the standard portion size (with the approximate equivalent size in grams) is as follows: slice of salted fish (70 g); 1 whole dried fish (50 g); quarter of a can of tuna (20 g); slice of salmon or trout (70 g); 4 sashimi slices of bonito or tuna (60 g); 4 sashimi slices of Japanese amberjack (60 g); half slice of cod or flatfish (40 g); slice of sea bream (70 g); 1 whole horse mackerel or sardine (80 g); 1 whole saury or mackerel (80 g); 2 tablespoons of small dried fish (10 g); one-quarter of a clutch of salted roe (20 g); half skewer of eel (50 g); 3 sashimi slices of squid (50 g); one-third of an octopus tentacle (50 g); 2 Chinese white shrimps (40 g); 10 shucked clams (20 g); 10 shucked pond snails (20 g); and fish paste products comprising one-sixth of chikuwa (20 g); 2 slices of kamaboko (20 g), and one-quarter of satsuma-age (20 g). The 9 frequency categories for each item were <1 time/mo, 1–3 times/mo, 1–2 times/wk, 3–4 times/wk, 5–6 times/wk, every day, 2–3 times/d, 4–6 times/d, and ≥7 times/d. Daily intake of fish (grams per day) was calculated as the frequency of consumption multiplied by the standard portion size for each fish item. The fatty acid composition table of Japanese foods (22) was used to calculate the daily intake of n–3 and n–6 PUFAs (data for the individual subtypes of fatty acids are not included in the JECS dataset). We performed log-transformation of fish and PUFA intake and calculated the energy-adjusted intake using the residual model (23). Because there were 4430 individuals for the 6-mo analysis and 4148 for the 1-y analysis whose maternal fish intake was 0 g/d, we replaced this value with 0.03 g/d, which is one-tenth of the lowest fish intake (0.3 g/d) of the participants (excluding 0 g/d). We did the same for 217 individuals for the 6-mo analysis and 189 for the 1-y analysis whose maternal n–3 PUFA intake was 0 g/d, replacing it with 0.001 g/d, which is one-tenth of the lowest n–3 PUFA intake of the participants (excluding 0 g/d).

### Psychomotor development

The quantitative use of the ASQ-3, a parent-completed screening method for monitoring children at risk of developmental delay, has been validated in epidemiological studies (24). The ASQ-3 comprises 21 age-specific structured questions divided among 5 domains: communication, gross motor skills, fine motor skills, problem-solving, and personal-social skills (24). Participants were administered the ASQ-3 at age 6 mo and 1 y. In total, the questionnaire comprises 30 questions (6 per domain) that can be answered with either “yes” (= 10), “sometimes” (= 5), or “not yet” (= 0), resulting in a score of 0–60 for each domain. If there were 1 or 2 missing answers out of the 6 questions, the remaining total score was multiplied by a correction coefficient of 1.2 or 1.5, respectively, to adjust the score to 0–60. If >2 of the 6 questions were not answered, the participant was excluded from the analysis. The timings of administering the 2 tools (at 6 mo and 1 y) were set to within a window period of ±1 mo (from 5 mo 0 d to 6 mo 30 d for the 6-mo analysis, and from 11 mo 0 d to 12 mo 30 d for the 1-y analysis); participants to whom the ASQ-3 was administered outside these periods were excluded. Age was corrected for prematurity if a child was born ≥3 wk before the due date. There are 2 items that ask about behaviors at 1 y of age that the child might have performed at one time but no longer does because he or she has acquired more advanced skills (one in the fine motor domain and another in the problem-solving domain). If parents answered “not yet” or “sometimes” to an easier item but “yes” to a more advanced item, the response for the earlier item was changed to “yes” (24). The screen-positive cases for each area are defined as those with scores on or below the respective threshold values, which are set at 2 SDs below the mean (i.e., *z*-score at or below −2) to yield a sensitivity of 85–92%, specificity of 78–92%, and positive predictive value of 32–64% (24).

### Statistical analysis

Unless stated otherwise, data are expressed as mean ± SD or median. We categorized participants according to quintile for fish or PUFA intake to estimate the risk of a participant being on or below the respective threshold (2 SD below the mean) of each domain of development for each level of fish intake and PUFA intake. We chose this 5-group approach because the previous largest study adopted quintiles (5). We then calculated ORs and 95% CIs using logistic regression analysis. In tests for trend, we assigned categorical numbers to the quintile distributions for fish intake and PUFA intake and evaluated them as continuous variables. We included potential confounding factors and covariates in the statistical analysis if previous studies found them to be associated (or are theoretically inferred to be associated) with the outcome. These were assessed by questionnaires administered during early and mid–late pregnancy. Because birth weight, gestation length, and breastfeeding are postexposure covariates and are considered mediators not confounders, we did not adjust for these covariates. The confounding factors and covariates used in this study were as follows: age; previous deliveries (nulliparous or multiparous); prepregnancy BMI (kg/m^2^) categorized as <18.5, 18.5–25, or ≥25); highest maternal educational level (1, junior high or high school; 2, technical junior college, technical/vocational college, associate degree; 3, bachelor's degree, postgraduate degree); annual household income (<4 million, 4–6 million, or ≥6 million JPY); marital status [1, married (including common-law status); 2, single (never married); 3, divorced or widowed]; alcohol intake (1, never; 2, previously drank alcohol but quit before learning of the pregnancy; 3, previously drank alcohol but quit after learning of the pregnancy; or 4, currently drinking; 5 participants were misclassified to a category No. 5 “quit,” which does not exist, so we treated them as having missing data and later imputed them using multiple imputation); smoking status (1, never smoked; 2, previously smoked but quit before learning of current pregnancy; 3, previously smoked but quit after learning of current pregnancy; 4, currently smoking); physical activity (25, 26) (MET, metabolic equivalent of a task measured as the number of minutes per day); employment status (yes or no); child's sex; presence of congenital anomaly (yes or no); and use of EPA and/or DHA supplementation (yes for ≥1–3 uses/wk or no for ≤2–3 uses/mon).

We performed multiple imputations for the missing values of covariates by using chained equations (27) to obtain 5 imputed datasets. Data were imputed differently according to time point (i.e., at age 6 mo and 1 y). We included auxiliary variables related to covariates to preserve the assumption of missing at random. Statistical significance was set at a 2-sided *P* value of <0.05. Analyses were performed with SAS version 9.4 (SAS Institute Inc).

## Results

### Participant characteristics

**Table 1** shows the maternal characteristics of the participants according to quintile for fish intake. Compared with those with low reported fish consumption, women with higher fish consumption were slightly older and more likely to be multiparous, have a higher education level and annual household income, be a current drinker, be a nonsmoker, and be more physically active. The associations were similar for dietary n–3 PUFA intake. As for a possible selection bias, of the 104,065 records at baseline, 22,368 records were not used for the 6-mo analysis due to the exclusion criteria described in the Study population section. Compared with nonparticipants, participants were more likely to be older, multiparous, educated, a nonsmoker, and employed and to have higher income. There were no material differences in the other covariates.

**TABLE 1:** Characteristics according to quintile for fish intake during pregnancy in women (*n* = 81,697)

	Quintile for fish intake				
	1 (low)	2	3	4	5 (high)
Median intake of fish,^1^ g/d	5.4	18.6	29.9	43.5	69.3
Age at delivery, y	30.6	31.2	31.4	31.7	31.5
Previous deliveries, *n* (%)					
Nullipara	7651 (46.8)	6789 (41.6)	6537 (40.0)	6420 (39.3)	6595 (40.4)
Multipara	8688 (53.2)	9551 (58.5)	9802 (60.0)	9920 (60.7)	9744 (59.6)
Prepregnancy BMI: kg/m^2^, *n* (%)					
<18.5	2683 (16.4)	2614 (16.0)	2571 (15.7)	2615 (16.0)	2656 (16.3)
18.5–25	11,965 (73.2)	12,126 (74.2)	12,228 (74.8)	12,036 (73.7)	11,924 (73.0)
≥25	1691 (10.4)	1600 (9.8)	1540 (9.4)	1689 (10.3)	1759 (10.8)
Highest educational level, *n* (%)					
Junior high school or high school	6734 (41.2)	5685 (34.8)	5384 (33.0)	5131 (31.4)	5625 (34.4)
Technical junior college, technical/vocationalcollege, or associate degree	6737 (41.2)	7101 (43.5)	7077 (43.3)	7088 (43.4)	6765 (41.4)
Bachelor's degree, postgraduate degree	2868 (17.6)	3554 (21.8)	3879 (23.7)	4121 (25.2)	3948 (24.2)
Annual household income (JPY), *n* (%)					
<4 million	7484 (45.8)	6586 (40.3)	6290 (38.5)	5975 (36.6)	6025 (36.9)
4–6 million	5137 (31.4)	5446 (33.3)	5555 (34.0)	5509 (33.7)	5568 (34.1)
≥6 million	3718 (22.8)	4309 (26.4)	4493 (27.5)	4856 (29.7)	4746 (29.1)
Marital status, *n* (%)					
Married (including common-law marriage)	15,395 (94.2)	15,707 (96.1)	15,783 (96.6)	15,808 (96.7)	15,708 (96.1)
Single (never married)	763 (4.7)	515 (3.2)	471 (2.9)	436 (2.7)	515 (3.2)
Divorced or widowed	181 (1.1)	118 (0.7)	86 (0.5)	97 (0.6)	115 (0.7)
Alcohol intake, *n* (%)					
Never	5556 (34.0)	5391 (33.0)	5529 (33.8)	5333 (32.6)	5565 (34.1)
Did previously but quit before learning of pregnancy	2749 (16.8)	2772 (17.0)	2718 (16.6)	2908 (17.8)	2776 (17.0)
Did previously but quit after learning of pregnancy	7635 (46.7)	7686 (47.0)	7618 (46.6)	7609 (46.6)	7572 (46.3)
Current	398 (2.4)	492 (3.0)	474 (2.9)	491 (3.0)	426 (2.6)
Smoking status, *n* (%)					
Never	8811 (53.9)	9568 (58.6)	9903 (60.6)	10106 (61.9)	9878 (60.5)
Did previously but quit before learning of pregnancy	3984 (24.4)	3997 (24.5)	3872 (23.7)	3825 (23.4)	3815 (23.4)
Did previously but quit after learning of pregnancy	2685 (16.4)	2151 (13.2)	1949 (11.9)	1872 (11.5)	2048 (12.5)
Currently smoking	859 (5.3)	623 (3.8)	615 (3.8)	537 (3.3)	597 (3.7)
Median physical activity: METs,^2^ min/d	70.7	70.7	70.7	70.7	74.0
Employed, *n* (%)	9287 (56.8)	8963 (54.9)	8857 (54.2)	8719 (53.4)	8581 (52.5)
Child's sex: boys, *n* (%)	8306 (50.8)	8365 (51.2)	8343 (51.1)	8326 (51.0)	8288 (50.7)
Congenital anomaly, *n* (%)	351 (2.2)	347 (2.1)	315 (1.9)	327 (2.0)	357 (2.2)
Use of EPA and/or DHA supplementation: yes, *n* (%)	434 (2.7)	408 (2.5)	390 (2.4)	403 (2.5)	464 (2.8)

1 Dietary intake between learning of pregnancy and the second/third trimester. Quintile medians in grams per day adjusted energy intake using the residual method.

2 MET, metabolic equivalent of a task.

### Scores for each psychomotor development domain and the respective threshold values

Scores (mean ± SD) for the 5 psychomotor development domains at age 6 mo were 46.6 ± 8.9 for communication, 33.5 ± 12.8 for gross motor, 40.9 ± 14.1 for fine motor, 44.1 ± 12.7 for problem-solving, and 34.3 ± 15.2 for personal-social. At age 1 y, the scores were 37.8 ± 13.4, 42.9 ± 17.5, 48.3 ± 11.6, 42.4 ± 13.6, and 37.1 ± 14.5, respectively. The respective threshold values were calculated by subtracting 2 SDs from the mean for each domain (24): they were 28.9, 7.9, 12.7, 18.8, and 4.0 at 6 mo, and 10.9, 7.8, 25.0, 15.2, and 8.1 at 1 y.

### Multivariable logistic regression for scoring at or below −2 SD of the mean for each psychomotor development domain

**Table 2** shows the multivariable ORs (with 95% CIs) for scoring at or below −2 SD of the mean for each psychomotor development domain at age 6 mo according to quintile for fish intake during pregnancy (*n* = 81,697). Reduced risk of delay was found in the fifth quintile for problem-solving at 6 mo. A trend test also revealed a significant linear association between fish consumption and problem-solving at 6 mo. At 1 y (**Table 3**), reduced risk of delay was found in the fifth quintile for fine motor and in the fourth and fifth quintiles for problem-solving, with trend tests also revealing significant linear associations. The significantly reduced risk of a 2 SD decline in these domains (*P* < 0.05) was mostly ∼10% compared with the reference group (the lowest group). Given that the prevalence of a 2 SD decline is 2.5%, reducing the risk of a 2 SD decline by 10% would correspond to 0.25% (2.5% × 10%) overall reduction. If we extrapolate this (0.25%) to all infants born in Japan in a 1-y period, which is ∼1 million, that would be 2500 infants, indicating 500 infants in each quintile (0.25%/quintile = 0.05%) would be prevented from neurodevelopmental delay. **Table 4** shows the summary of adjusted ORs for dietary intake of fish and PUFAs according to psychomotor development domain. Details for fish intake are shown in Tables 2 and 3, n–3 PUFAs in **Supplemental Tables 1** and **2**, n–6 PUFAs in **Supplemental Tables 3** and **4**, and n–6/n–3 PUFAs in **Supplemental Tables 5** and **6**.

**TABLE 2:** For 6-mo-old infants, ORs (95% CIs) of scores at or below −2 SDs of the mean for each development domain according to quintile for fish intake during pregnancy (*n* = 81,697)

	Quintile for fish intake					*P* value for trend
	1 (low)	2	3	4	5 (high)	
Median intake of fish,^1^ g/d	5.4	18.6	29.9	43.5	69.3	
Communication						
Scores >2 SDs below average	16,032	16,003	16,017	16,013	16,021	
Scores ≤2 SDs below average	307	337	322	327	318	
Crude OR	1.00	1.10 (0.94, 1.29)	1.05 (0.90, 1.23)	1.07 (0.91, 1.25)	1.04 (0.89, 1.21)	0.8
Adjusted OR^2^	1.00	1.02 (0.87, 1.19)	0.95 (0.81, 1.11)	0.94 (0.81, 1.11)	0.93 (0.79, 1.09)	0.2
Gross motor						
Scores >2 SDs below average	16,061	16,015	16,040	16,051	16,057	
Scores ≤2 SDs below average	278	325	299	289	282	
Crude OR	1.00	1.17 (0.997, 1.38)	1.08 (0.91, 1.27)	1.04 (0.88, 1.23)	1.02 (0.86, 1.20)	0.6
Adjusted OR^2^	1.00	1.13 (0.96, 1.33)	1.03 (0.87, 1.21)	0.98 (0.83, 1.16)	0.96 (0.81, 1.13)	0.2
Fine motor						
Scores >2 SDs below average	15,892	15,918	15,887	15,897	15,885	
Scores ≤2 SDs below average	447	422	452	443	454	
Crude OR	1.00	0.94 (0.82, 1.08)	1.01 (0.89, 1.16)	0.99 (0.87, 1.13)	1.02 (0.89, 1.16)	0.6
Adjusted OR^2^	1.00	0.86 (0.75, 0.99)	0.90 (0.78, 1.03)	0.86 (0.75, 0.98)	0.90 (0.78, 1.02)	0.2
Problem-solving						
Scores >2 SDs below average	15,690	15,667	15,704	15,690	15,714	
Scores ≤2 SDs below average	649	673	635	650	625	
Crude OR	1.00	1.04 (0.93, 1.16)	0.98 (0.87, 1.09)	1.00 (0.90, 1.12)	0.96 (0.86, 1.08)	0.4
Adjusted OR^2^	1.00	0.98 (0.88, 1.09)	0.90 (0.81, 1.01)	0.91 (0.81, 1.02)	0.88 (0.79, 0.99)	0.01
Personal-social						
Scores >2 SDs below average	16,120	16,127	16,103	16,114	16,095	
Scores ≤2 SDs below average	219	213	236	226	244	
Crude OR	1.00	0.97 (0.80, 1.18)	1.08 (0.90, 1.30)	1.03 (0.86, 1.25)	1.12 (0.93, 1.34)	0.2
Adjusted OR^2^	1.00	0.91 (0.75, 1.10)	0.98 (0.82, 1.19)	0.92 (0.76, 1.11)	1.01 (0.84, 1.22)	0.8

1 Dietary intake between learning of pregnancy and the second/third trimester. Quintile medians in grams per day adjusted energy intake using the residual method.

2 Covariates were adjusted for age, previous deliveries, prepregnancy BMI, highest maternal educational level, annual household income, marital status, alcohol intake, smoking status, physical activity, employment status, presence of congenital anomaly, child's sex, and use of EPA and/or DHA supplementation.

**TABLE 3:** For 1-y-old infants, ORs (95% CIs) of scores at or below −2 SDs of the mean for each development domain according to quintile for fish intake during pregnancy (*n* = 77,751)

	Quintile for fish intake					*P* value for trend
	1 (low)	2	3	4	5 (high)	
Median intake of fish,^1^ g/d	5.5	18.6	30.0	43.5	69.1	
Communication						
Scores >2 SDs below average	15,062	15,014	15,003	15,032	15,020	
Scores ≤2 SDs below average	488	536	548	518	530	
Crude OR	1.00	1.10 (0.97, 1.25)	1.13 (0.996, 1.28)	1.06 (0.94, 1.21)	1.09 (0.96, 1.23)	0.4
Adjusted OR^2^	1.00	1.05 (0.93, 1.19)	1.06 (0.93, 1.20)	0.98 (0.86, 1.11)	1.00 (0.88, 1.14)	0.6
Gross motor						
Scores >2 SDs below average	14,683	14,674	14,619	14,593	14,646	
Scores ≤2 SDs below average	867	876	932	957	904	
Crude OR	1.00	1.01 (0.92, 1.11)	1.08 (0.98, 1.19)	1.11 (1.01, 1.22)	1.05 (0.95, 1.15)	0.09
Adjusted OR^2^	1.00	0.97 (0.88, 1.07)	1.03 (0.94, 1.13)	1.04 (0.95, 1.15)	0.98 (0.89, 1.08)	0.8
Fine motor						
Scores >2 SDs below average	14,603	14,620	14,581	14,633	14,659	
Scores ≤2 SDs below average	947	930	970	917	891	
Crude OR	1.00	0.98 (0.89, 1.08)	1.03 (0.94, 1.13)	0.97 (0.88, 1.06)	0.94 (0.85, 1.03)	0.2
Adjusted OR^2^	1.00	0.95 (0.87, 1.05)	0.99 (0.90, 1.08)	0.92 (0.84, 1.01)	0.90 (0.81, 0.99)	0.02
Problem-solving						
Scores >2 SDs below average	14,688	14,693	14,699	14,756	14,747	
Scores ≤2 SDs below average	862	857	852	794	803	
Crude OR	1.00	0.99 (0.90, 1.10)	0.99 (0.90, 1.09)	0.92 (0.83, 1.01)	0.93 (0.84, 1.02)	0.04
Adjusted OR^2^	1.00	0.98 (0.89, 1.08)	0.97 (0.88, 1.07)	0.89 (0.81, 0.98)	0.90 (0.81, 0.99)	0.005
Personal-social						
Scores >2 SDs below average	15,086	15,097	15,096	15,039	15,056	
Scores ≤2 SDs below average	464	453	455	511	494	
Crude OR	1.00	0.98 (0.86, 1.11)	0.98 (0.86, 1.12)	1.11 (0.97, 1.26)	1.07 (0.94, 1.21)	0.08
Adjusted OR^2^	1.00	0.92 (0.81, 1.05)	0.91 (0.80, 1.04)	1.01 (0.89, 1.15)	0.99 (0.87, 1.12)	0.6

1 Dietary intake between learning of pregnancy and the second/third trimester. Quintile medians in grams per day adjusted energy intake using the residual method.

2 Covariates were adjusted for age, previous deliveries, prepregnancy BMI, highest maternal educational level, annual household income, marital status, alcohol intake, smoking status, physical activity, employment status, presence of congenital anomaly, child's sex, and use of EPA and/or DHA supplementation.

**TABLE 4:** Summary of adjusted ORs for dietary intake of fish and PUFAs for each development domain^1^

	Assessment at 6 mo (*n* = 81,697)						Assessment at 1 y (*n* = 77,751)					
	Quintile for fish or PUFA intake					*P* value for trend	Quintile for fish or PUFA intake					*P* value for trend
	1 (low)	2	3	4	5 (high)		1 (low)	2	3	4	5 (high)	
Fish^2^												
Communication	1.00	1.02	0.95	0.94	0.93	0.2	1.00	1.05	1.06	0.98	1.00	0.6
Gross motor	1.00	1.13	1.03	0.98	0.96	0.2	1.00	0.97	1.03	1.04	0.98	0.8
Fine motor	1.00	0.86*	0.90	0.86*	0.90	0.2	1.00	0.95	0.99	0.92	0.90*	0.02
Problem-solving	1.00	0.98	0.90	0.91	0.88*	0.01	1.00	0.98	0.97	0.89*	0.90*	0.005
Personal-social	1.00	0.91	0.98	0.92	1.01	0.8	1.00	0.92	0.91	1.01	0.99	0.6
n–3 PUFAs^2^												
Communication	1.00	0.86	1.03	0.94	0.84*	0.2	1.00	1.00	1.00	0.97	0.93	0.2
Gross motor	1.00	1.16	0.97	1.01	1.03	0.6	1.00	0.95	1.00	0.93	0.93	0.2
Fine motor	1.00	0.91	0.88	0.84*	0.87*	0.02	1.00	0.92	0.92	0.90*	0.86**	0.003
Problem-solving	1.00	0.91	0.95	0.89*	0.89*	0.053	1.00	0.90*	0.87**	0.88*	0.79***	<0.0001
Personal-social	1.00	0.89	0.97	1.01	1.00	0.6	1.00	0.85*	0.91	0.91	0.96	0.9
n–6 PUFAs^2^												
Communication	1.00	1.06	0.92	1.04	0.83*	0.04	1.00	1.06	0.97	1.00	1.01	0.8
Gross motor	1.00	1.06	0.98	1.02	0.94	0.4	1.00	0.96	0.93	0.88**	0.91*	0.008
Fine motor	1.00	0.99	0.87*	0.90	0.84*	0.003	1.00	0.95	0.91*	0.95	0.89*	0.04
Problem-solving	1.00	0.96	0.90	0.96	0.90	0.104	1.00	0.88**	0.88*	0.87**	0.84***	0.001
Personal-social	1.00	0.93	0.83	1.02	0.95	1.0	1.00	0.91	0.89	0.94	0.98	0.9
n–6/n–3 ratios^2^												
Communication	1.00	1.12	1.04	1.07	1.09	0.5	1.00	0.99	1.02	0.95	1.07	0.5
Gross motor	1.00	1.02	1.03	0.96	1.08	0.6	1.00	0.99	1.02	0.87**	1.05	0.9
Fine motor	1.00	0.97	0.99	0.94	1.10	0.3	1.00	0.98	1.06	0.97	1.09	0.103
Problem-solving	1.00	1.09	1.04	1.02	1.14*	0.12	1.00	0.99	1.05	1.01	1.13*	0.02
Personal-social	1.00	1.06	0.86	0.97	0.98	0.5	1.00	0.99	0.97	0.97	1.00	0.8

1 ORs and *P* values for trend were calculated by multivariable logistic regression analysis adjusted for age, previous deliveries, prepregnancy BMI, highest maternal educational level, annual household income, marital status, alcohol intake, smoking status, physical activity, employment status, presence of congenital anomaly, child's sex, and use of EPA and/or DHA supplementation. *P<0.05, **P<0.01, ***P<0.001.

Details of fish are described in Tables 2 and 3, n–3 PUFAs in Supplemental Tables 1 and 2, n–6 PUFAs in Supplemental Tables 3 and 4, and n–6/n–3 PUFAs in Supplemental Tables 5 and 6.

2 Dietary intake between learning of pregnancy and the second/third trimester.

Supplemental Tables 1 and 2 show the multivariable ORs and 95% CIs at 6 mo and 1 y, respectively, according to quintile for n–3 PUFA intake during pregnancy. Significantly reduced risk of delay was observed in some quintiles for communication, fine motor, and problem-solving. A trend test also revealed a significant linear association between n–3 PUFA consumption and fine motor at 6 mo. In the analysis of 1-y-olds, the significant association disappeared in communication whereas a significant association emerged in the personal domain. The largest risk reduction was in problem-solving in the fifth quintile (OR = 0.79; 95% CI: 0.71, 0.87). Trend tests revealed significant linear associations of n–3 PUFA consumption with fine motor and problem-solving.

Supplemental Tables 3 and 4 show the multivariable ORs and 95% CIs for age 6 mo and 1 y, respectively, according to quintile for n–6 PUFA intake during pregnancy. Significantly reduced risk of delay was observed in some quintiles for communication and fine motor at 6 mo, with a trend test revealing a significant linear association between n–6 PUFA consumption and fine motor. However, at 1 y, the significant risk reduction had disappeared in communication and emerged in gross motor and problem-solving. Trend tests revealed significant linear associations of n–6 PUFA consumption with gross motor, fine motor, and problem-solving.

Supplemental Tables 5 and 6 show the multivariable ORs and 95% CIs for 6 mo and 1 y, respectively, according to quintile for n–6/n–3 ratio intake during pregnancy. There was significantly increased risk in the fifth quintiles for problem-solving at both 6 mo and 1 y, with a trend test revealing a significant linear association between the n–6/n–3 ratio and problem-solving at 1 y. Also, there was a significantly reduced risk of delay in the fourth quintile for gross motor at 1 y.

## Discussion

Maternal fish intake during pregnancy was independently associated with reduced risk of delay in some domains of child psychomotor development (mainly in fine motor and problem-solving) at age 6 mo and 1 y. Dietary intake of total n–3 and total n–6 PUFAs was also associated with a reduced risk in some domains. In contrast, the dietary n–6/n–3 PUFA ratio was associated with increased risk of delay in problem-solving. At the same time, however, we need to acknowledge the large number of nonsignificant associations that exist.

The findings of randomized controlled trials (RCTs) using fish oil and/or n–3 PUFAs are inconsistent (28, 29), whereas many observational studies have shown benefits (1–10) but not all (11, 12). In general, RCTs provide a higher level of evidence than observational studies, although RCTs have some limitations, such as being unable to actively reduce nutrient intake due to ethical reasons, not being suitable for evaluating long-term interventions, and not being able to intervene with the consumption of fish as a whole food. In these aspects, observational studies have the advantage; they can compare between extremely low and high consumption of nutrients and are suitable for long-term follow-up, enabling us to investigate the effects of nutrient intake in utero over the long term, which is important to consider in the developmental origin of health and disease (DOHaD) theory. We have previously reported that PUFA deficiency during the early neurodevelopmental period in mice could model the prodromal state of schizophrenia through changes in the epigenetic regulation (DOHaD theory) of nuclear receptor genes (30). Other animal studies have indicated that epigenetic phenomena could be an important mechanism underlying the DOHaD theory and that RCTs with nutritional interventions might need to be started preconceptionally (31). Conducting this kind of RCT is essentially unrealistic, giving observational studies the advantage in this case. Consuming fish as a whole food might offer synergistic effects of nutrients (32) because fish is a good source of nutrients that are important for infant development such as iodine and vitamins A, D, and B-12 (33). Nevertheless, a disadvantage of observational studies is that the associations observed could be attributable to confounding by unmeasured confounding variables and/or residual confounding, which is a limitation of the present study by nature of its design.

Besides DHA, arachidonic acid (AA) is also an important PUFA. It is abundant in the gray matter [∼8–10% for AA and 9–14% for DHA (34, 35)] and is a required precursor for eicosanoids such as PGs, thromboxane, and leukotriene, which are potent regulatory and inflammatory substances (36). AA also showed neuroprotective effects in an animal study (37) and was involved in the growth of neurites in a cell culture study (38). Our results showed that the effects of n–6 PUFAs on neurodevelopment (Supplemental Tables 3 and 4) were similar to those of n–3 PUFAs (Supplemental Tables 1 and 2). However, for the n–6/n–3 ratio, detrimental effects emerged for the problem-solving domain in the highest quintile group (median ratio = 7.28–7.29; Supplemental Tables 5 and 6). If we take a closer look at the ORs for both the n–6/n–3 ratio and n–6 at 1 y of age in the problem-solving domain where there is a significant trend, only the highest quintile for the n–6/n–3 ratio showed a significant increased risk; n–6 showed a reduced risk from the second through to the highest quintile, and the ORs are similar (in other words, the lowest quintile for n–6 showed the increased risk). This indicates that there might be a minimum requirement of n–6 for the prevention of problem-solving delay, and at the same time the association of the excess of n–6 relative to n–3 (more specifically, n–6/n–3 ratio = 7.29) with detrimental effects. The reason for this is unclear, but it possibly involves the association of n–6 and n–3 PUFAs with preterm birth, which is a known risk factor for neurodevelopmental delay (39). A case-control study revealed that maternal AA in blood was increased in preterm cases compared with controls at delivery, and EPA and n–3/n–6 ratios were lower (in other words, the n–6/n–3 ratios were higher) in preterm cases than in controls at delivery (40). Because AA-derived PG is known to initiate delivery, the presence of n–3 PUFAs might act as a competitive inhibitor of AA and consequently reduce the risk of preterm birth (41). Our findings of this detrimental effect of the n–6/n–3 ratio are consistent with those of a French study (among never-breastfed children) (42) and those of a Korean study (12), but not with those of a Japanese study (18). Because the discrepancies might be due to when the neurodevelopment was assessed (at age 2–3 y, 6 mo, and 5 y, respectively) and to the type of measurement applied, future work requires a longer follow-up period and needs to additionally measure behavioral problems.

One of the main strengths of this study is its large sample size, with the study conducted nationwide in 15 community-based centers in Japan; the participants are thus representative of pregnant women in the community. In addition, the ASQ-3 used in this study as a screening tool for monitoring children who are at risk of developmental delay is well validated for quantitative use in epidemiological studies and is used worldwide (24). The prospective data collection of exposures and outcomes minimizes recall bias, and almost all of the important confounders were included in the model. Moreover, the FFQ contains as many as 21 items related to fish or shellfish consumption (21).

There are also, however, some limitations of this study to consider. First, use of the FFQ has not been validated in pregnant women. Second, as mentioned earlier, due to the observational nature of the study, the results could have been confounded by unmeasured residual factors. For instance, fish intake might function as a proxy for an overall healthy lifestyle (43). Third, the dataset did not consider n–3 PUFA subtypes (e.g., EPA, DHA, α-linolenic acid, and docosapentaenoic acid). Fourth, the ASQ-3 findings are based on parental report, which raises a concern about subjectivity on the part of the parents, possibly inflating positive effects. Fifth, only 4 smoking statuses were available during pregnancy (1, never smoked; 2, previously smoked but quit before learning of current pregnancy; 3, previously smoked but quit after learning of current pregnancy; and 4, currently smoking). If data of the amount actually smoked were available instead, the association between n–3 PUFAs and neurodevelopment might have been clearer because the amount could affect PUFAs through degradation (44). Sixth, because rapid and dynamic brain development continues until 2 y of age (45), postnatal dietary exposure should also have been taken into consideration. Seventh, because the participants in this study are a representative sample of Japanese pregnant women whose fish consumption is high compared with their Western counterparts, the present findings might not be generalizable to Western populations. Lastly, due to multiple comparison, it is likely that some of the observed differences could be due to chance.

In terms of clinical implications, the evidence supporting the efficacy of n–3 PUFAs in child neurodevelopment is still inconsistent (28, 29). However, because there is considerable evidence of the beneficial effects of fish consumption on neurodevelopment, we advocate that fish—especially fatty fish (10)—be recommended to pregnant women, as long as the fish species are limited to those not containing methylmercury. In terms of research implications, because impaired early communication skills can be recognizable as symptoms of autism even in the first year of life (46, 47), more follow-up is needed to explore other aspects of neurodevelopment such as mental health and behavior.

To conclude, dietary intake of fish and PUFAs (both n–3 and n–6) in pregnancy was partly associated with a reduced risk of suboptimal neurodevelopment outcomes at age 6 mo and 1 y. The dietary n–6/n–3 ratio was positively associated with increased risk for 1 neurodevelopment domain. The present findings should be interpreted with caution, and future study is needed to focus on older children to examine if the beneficial effects persist.

## Supplementary Material

nqaa190_Supplemental_Tables

## Notes

The JECS was funded by the Ministry of the Environment, Japan. The findings and conclusions of this article are solely the responsibility of the authors and do not represent the official views of the Ministry of the Environment, Japan.

Data described in the manuscript, code book, and analytic code will not be made available due to ethical restrictions and the specific legal framework in Japan. The Ethical Guidelines for Epidemiological Research issued by the Japan Ministry of Education, Culture, Sports, Science, and Technology and the Ministry of Health, Labour, and Welfare restrict the open sharing of epidemiological data.

Supplemental Tables 1–6 are available from the “Supplementary data” link in the online posting of the article and from the same link in the online table of contents at https://academic.oup.com/ajcn/.

Abbreviations used: AA, arachidonic acid; ASQ, Ages & Stages Questionnaire; DOHaD, developmental origin of health and disease; JECS, Japan Environment and Children's Study; RCT, randomized controlled trial.
