# Supplementary material for: Maternal dietary intake of fish and PUFAs and child neurodevelopment at 6 months and 1 year of age: a nationwide birth cohort—the Japan Environment and Children's Study (JECS)
Source: Am J Clin Nutr. 2020 Aug 7;112(5):1295–303. doi: 10.1093/ajcn/nqaa190 (PMC7657336; doi:10.1093/ajcn/nqaa190)
Supplement: nqaa190_Supplemental_Tables [file nqaa190_supplemental_tables.docx]

Maternal dietary intake of fish and polyunsaturated fatty acids and child neurodevelopment at 6 months and 1 year of age: a nationwide birth cohort–the Japan Environment and Children’s Study

Kei Hamazaki

On-line Supplementary Material

**Supplementary Table 1.** For 6-month-old infants, ORs (95% CIs) of scores at or below -2 SDs of the mean for each development domain according to quintile for n-3 PUFA intake during pregnancy (n = 81,697).

|  | Quintile for n-3 PUFA intake | | | | | *p* value |
| --- | --- | --- | --- | --- | --- | --- |
|  | 1 (low) | 2 | 3 | 4 | 5 (high) | for trend |
| **Median intake of n-3 PUFAs, g/day ^1^** | 1.09 | 1.47 | 1.74 | 2.03 | 2.55 |  |
| **Communication** |  |  |  |  |  |  |
| Scores >2SDs below average | 16,009 | 16,044 | 15,982 | 16,007 | 16,044 |  |
| Scores ≤2SDs below average | 330 | 296 | 357 | 333 | 295 |  |
| Crude OR | 1.00 | 0.90 (0.76, 1.05) | 1.08 (0.93, 1.26) | 1.01 (0.87, 1.18) | 0.89 (0.76, 1.05) | 0.6 |
| Adjusted OR ^2^ | 1.00 | 0.86 (0.74, 1.01) | 1.03 (0.89, 1.20) | 0.94 (0.80, 1.09) | **0.84 (0.72, 0.99)** | 0.2 |
| **Gross Motor** |  |  |  |  |  |  |
| Scores >2SDs below average | 16,060 | 16,013 | 16,062 | 16,046 | 16,043 |  |
| Scores ≤2SDs below average | 279 | 327 | 277 | 294 | 296 |  |
| Crude OR | 1.00 | **1.18 (1.00, 1.38)** | 0.99 (0.84, 1.17) | 1.06 (0.89, 1.24) | 1.06 (0.90, 1.25) | 1.0 |
| Adjusted OR ^2^ | 1.00 | 1.16 (0.99, 1.37) | 0.97 (0.82, 1.15) | 1.01 (0.86, 1.20) | 1.03 (0.87, 1.21) | 0.6 |
| **Fine Motor** |  |  |  |  |  |  |
| Scores >2SDs below average | 15,871 | 15,894 | 15,902 | 15,910 | 15,902 |  |
| Scores ≤2SDs below average | 468 | 446 | 437 | 430 | 437 |  |
| Crude OR | 1.00 | 0.95 (0.83, 1.09) | 0.93 (0.82, 1.06) | 0.92 (0.80, 1.05) | 0.93 (0.82, 1.06) | 0.2 |
| Adjusted OR ^2^ | 1.00 | 0.91 (0.80, 1.04) | 0.88 (0.77, 1.00) | **0.84 (0.73, 0.96)** | **0.87 (0.76, 0.99)** | **0.02** |
| **Problem Solving** |  |  |  |  |  |  |
| Scores >2SDs below average | 15,667 | 15,712 | 15,674 | 15,703 | 15,709 |  |
| Scores ≤2SDs below average | 672 | 628 | 665 | 637 | 630 |  |
| Crude OR | 1.00 | 0.93 (0.83, 1.04) | 0.99 (0.89, 1.10) | 0.95 (0.85, 1.06) | 0.94 (0.84, 1.05) | 0.3 |
| Adjusted OR ^2^ | 1.00 | 0.91 (0.81, 1.02) | 0.95 (0.85, 1.06) | **0.89 (0.80, 1.00)** | **0.89 (0.80, 1.00)** | 0.053 |
| **Personal-Social** |  |  |  |  |  |  |
| Scores >2SDs below average | 16,113 | 16,132 | 16,112 | 16,098 | 16,104 |  |
| Scores ≤2SDs below average | 226 | 208 | 227 | 242 | 235 |  |
| Crude OR | 1.00 | 0.92 (0.76, 1.11) | 1.00 (0.83, 1.21) | 1.07 (0.89, 1.29) | 1.04 (0.87, 1.25) | 0.3 |
| Adjusted OR ^2^ | 1.00 | 0.89 (0.74, 1.08) | 0.97 (0.80, 1.17) | 1.01 (0.84, 1.21) | 1.00 (0.83, 1.20) | 0.6 |

^1^ Dietary intake between learning of pregnancy and the second/third trimester. Quintile medians in g/day adjusted energy intake using the residual method.

^2^ Covariates were adjusted for age, previous deliveries, pre-pregnancy BMI, highest maternal educational level, annual household income, marital status, alcohol intake, smoking status, physical activity, employment status, presence of congenital anomaly, child’s sex, and use of EPA and/or DHA supplementation.

Odds ratios and *p* values for trend were calculated by logistic regression analysis. Values in bold indicate significance.

**Supplementary Table 2.** For 1-year-old infants, ORs (95% CIs) of scores at or below -2 SDs of the mean for each development domain according to quintile for n-3 PUFA intake during pregnancy (n=77,751).

|  | Quintile for n-3 PUFA intake | | | | | *p* value |
| --- | --- | --- | --- | --- | --- | --- |
|  | 1 (low) | 2 | 3 | 4 | 5 (high) | for trend |
| **Median intake of n-3 PUFAs, g/day^1^** | 1.09 | 1.47 | 1.74 | 2.03 | 2.54 |  |
| **Communication** |  |  |  |  |  |  |
| Scores >2SDs below average | 15,032 | 15,020 | 15,019 | 15,017 | 15,043 |  |
| Scores ≤2SDs below average | 518 | 530 | 532 | 533 | 507 |  |
| Crude OR | 1.00 | 1.02 (0.91, 1.16) | 1.03 (0.91, 1.16) | 1.03 (0.91, 1.17) | 0.98 (0.86, 1.11) | 0.8 |
| Adjusted OR ^2^ | 1.00 | 1.00 (0.88, 1.13) | 1.00 (0.88, 1.13) | 0.97 (0.86, 1.10) | 0.93 (0.82, 1.06) | 0.2 |
| **Gross Motor** |  |  |  |  |  |  |
| Scores >2SDs below average | 14,629 | 14,661 | 14,613 | 14,654 | 14,658 |  |
| Scores ≤2SDs below average | 921 | 889 | 938 | 896 | 892 |  |
| Crude OR | 1.00 | 0.96 (0.88, 1.06) | 1.02 (0.93, 1.12) | 0.97 (0.88, 1.07) | 0.97 (0.88, 1.06) | 0.6 |
| Adjusted OR ^2^ | 1.00 | 0.95 (0.86, 1.05) | 1.00 (0.91, 1.10) | 0.93 (0.85, 1.03) | 0.93 (0.85, 1.03) | 0.2 |
| **Fine Motor** |  |  |  |  |  |  |
| Scores >2SDs below average | 14,557 | 14,625 | 14,621 | 14,621 | 14,672 |  |
| Scores ≤2SDs below average | 993 | 925 | 930 | 929 | 878 |  |
| Crude OR | 1.00 | 0.93 (0.85, 1.02) | 0.93 (0.85, 1.02) | 0.93 (0.85, 1.02) | **0.88 (0.80, 0.96)** | **0.02** |
| Adjusted OR ^2^ | 1.00 | 0.92 (0.84, 1.01) | 0.92 (0.84, 1.01) | **0.90 (0.82, 0.99)** | **0.86 (0.78, 0.94)** | **0.003** |
| **Problem Solving** |  |  |  |  |  |  |
| Scores >2SDs below average | 14,623 | 14,710 | 14,741 | 14,712 | 14,797 |  |
| Scores ≤2SDs below average | 927 | 840 | 810 | 838 | 753 |  |
| Crude OR | 1.00 | **0.90 (0.82, 0.99)** | **0.87 (0.79, 0.96)** | **0.90 (0.82, 0.99)** | **0.80 (0.73, 0.89)** | **<0.0001** |
| Adjusted OR ^2^ | 1.00 | **0.90 (0.82, 0.99)** | **0.87 (0.79, 0.95)** | **0.88 (0.80, 0.97)** | **0.79 (0.71, 0.87)** | **<0.0001** |
| **Personal-Social** |  |  |  |  |  |  |
| Scores >2SDs below average | 15,053 | 15,114 | 15,083 | 15,072 | 15,052 |  |
| Scores ≤2SDs below average | 497 | 436 | 468 | 478 | 498 |  |
| Crude OR | 1.00 | **0.87 (0.77, 1.00)** | 0.94 (0.83, 1.07) | 0.96 (0.85, 1.09) | 1.00 (0.88, 1.14) | 0.5 |
| Adjusted OR ^2^ | 1.00 | **0.85 (0.75, 0.97)** | 0.91 (0.80, 1.04) | 0.91 (0.80, 1.03) | 0.96 (0.85, 1.09) | 0.9 |

^1^ Dietary intake between learning of pregnancy and the second/third trimester. Quintile medians in g/day adjusted energy intake using the residual method.

^2^ Covariates were adjusted for age, previous deliveries, pre-pregnancy BMI, highest maternal educational level, annual household income, marital status, alcohol intake, smoking status, physical activity, employment status, presence of congenital anomaly, child’s sex, and use of EPA and/or DHA supplementation.

Odds ratios and *p* values for trend were calculated by logistic regression analysis. Values in bold indicate significance.

**Supplementary Table 3.** For 6-month-old infants, ORs (95% CIs) of scores at or below -2 SDs of the mean for each development domain according to quintile for n-6 PUFA intake during pregnancy (n = 81,697).

|  | Quintile for n-6 PUFA intake | | | | | *p* value |
| --- | --- | --- | --- | --- | --- | --- |
|  | 1 (low) | 2 | 3 | 4 | 5 (high) | for trend |
| **Median intake of n-6 PUFAs, g/day ^1^** | 6.83 | 8.38 | 9.46 | 10.61 | 12.59 |  |
| **Communication** |  |  |  |  |  |  |
| Scores >2SDs below average | 16,013 | 15,985 | 16,029 | 15,992 | 16,067 |  |
| Scores ≤2SDs below average | 326 | 355 | 310 | 348 | 272 |  |
| Crude OR | 1.00 | 1.09 (0.94, 1.27) | 0.95 (0.81, 1.11) | 1.07 (0.92, 1.25) | **0.83 (0.71, 0.98)** | **0.04** |
| Adjusted OR ^2^ | 1.00 | 1.06 (0.91, 1.23) | 0.92 (0.79, 1.08) | 1.04 (0.89, 1.21) | **0.83 (0.71, 0.98)** | **0.04** |
| **Gross Motor** |  |  |  |  |  |  |
| Scores >2SDs below average | 16,047 | 16,026 | 16,048 | 16,039 | 16,064 |  |
| Scores ≤2SDs below average | 292 | 314 | 291 | 301 | 275 |  |
| Crude OR | 1.00 | 1.08 (0.92, 1.27) | 1.00 (0.85, 1.17) | 1.03 (0.88, 1.21) | 0.94 (0.80, 1.11) | 0.4 |
| Adjusted OR ^2^ | 1.00 | 1.06 (0.90, 1.25) | 0.98 (0.83, 1.16) | 1.02 (0.86, 1.20) | 0.94 (0.80, 1.11) | 0.4 |
| **Fine Motor** |  |  |  |  |  |  |
| Scores >2SDs below average | 15,867 | 15,858 | 15,911 | 15,900 | 15,943 |  |
| Scores ≤2SDs below average | 472 | 482 | 428 | 440 | 396 |  |
| Crude OR | 1.00 | 1.02 (0.90, 1.16) | 0.90 (0.79, 1.03) | 0.93 (0.82, 1.06) | **0.84 (0.73, 0.96)** | **0.003** |
| Adjusted OR ^2^ | 1.00 | 0.99 (0.87, 1.12) | **0.87 (0.76, 1.00)** | 0.90 (0.79, 1.03) | **0.84 (0.73, 0.96)** | **0.003** |
| **Problem Solving** |  |  |  |  |  |  |
| Scores >2SDs below average | 15,665 | 15,676 | 15,714 | 15,678 | 15,732 |  |
| Scores ≤2SDs below average | 674 | 664 | 625 | 662 | 607 |  |
| Crude OR | 1.00 | 0.98 (0.88, 1.10) | 0.92 (0.83, 1.03) | 0.98 (0.88, 1.10) | 0.90 (0.80, 1.00) | 0.08 |
| Adjusted OR ^2^ | 1.00 | 0.96 (0.86, 1.08) | 0.90 (0.81, 1.01) | 0.96 (0.86, 1.08) | 0.90 (0.81, 1.01) | 0.104 |
| **Personal-Social** |  |  |  |  |  |  |
| Scores >2SDs below average | 16,100 | 16,114 | 16,136 | 16,093 | 16,116 |  |
| Scores ≤2SDs below average | 239 | 226 | 203 | 247 | 223 |  |
| Crude OR | 1.00 | 0.95 (0.79, 1.14) | 0.85 (0.70, 1.02) | 1.03 (0.86, 1.24) | 0.93 (0.78, 1.12) | 0.8 |
| Adjusted OR ^2^ | 1.00 | 0.93 (0.77, 1.11) | 0.83 (0.69, 1.01) | 1.02 (0.85, 1.22) | 0.95 (0.79, 1.15) | 1.0 |

^1^ Dietary intake between learning of pregnancy and the second/third trimester. Quintile medians in g/day adjusted energy intake using the residual method.

^2^ Covariates were adjusted for age, previous deliveries, pre-pregnancy BMI, highest maternal educational level, annual household income, marital status, alcohol intake, smoking status, physical activity, employment status, presence of congenital anomaly, child’s sex, and use of EPA and/or DHA supplementation.

Odds ratios and *p* values for trend were calculated by logistic regression analysis. Values in bold indicate significance.

**Supplementary Table 4.** For 1-year-old infants, ORs (95% CIs) of scores at or below -2 SDs of the mean for each development domain according to quintile for n-6 PUFA intake during pregnancy (n = 77,751).

|  | Quintile for n-6 PUFA intake | | | | | *p* value |
| --- | --- | --- | --- | --- | --- | --- |
|  | 1 (low) | 2 | 3 | 4 | 5 (high) | for trend |
| **Median intake of n-6 PUFAs, g/day^1^** | 6.84 | 8.39 | 9.47 | 10.61 | 12.57 |  |
| **Communication** |  |  |  |  |  |  |
| Scores >2SDs below average | 15,037 | 14,997 | 15,046 | 15,028 | 15,023 |  |
| Scores ≤2SDs below average | 513 | 553 | 505 | 522 | 527 |  |
| Crude OR | 1.00 | 1.08 (0.96, 1.22) | 0.98 (0.87, 1.12) | 1.02 (0.90, 1.15) | 1.03 (0.91, 1.16) | 1.0 |
| Adjusted OR ^2^ | 1.00 | 1.06 (0.93, 1.20) | 0.97 (0.85, 1.10) | 1.00 (0.88, 1.13) | 1.01 (0.89, 1.15) | 0.8 |
| **Gross Motor** |  |  |  |  |  |  |
| Scores >2SDs below average | 14,592 | 14,615 | 14,646 | 14,689 | 14,673 |  |
| Scores ≤2SDs below average | 958 | 935 | 905 | 861 | 877 |  |
| Crude OR | 1.00 | 0.97 (0.89, 1.07) | 0.94 (0.86, 1.03) | **0.89 (0.81, 0.98)** | 0.91 (0.83, 1.00) | **0.011** |
| Adjusted OR ^2^ | 1.00 | 0.96 (0.87, 1.05) | 0.93 (0.84, 1.02) | **0.88 (0.80, 0.97)** | **0.91 (0.82, 1.00)** | **0.008** |
| **Fine Motor** |  |  |  |  |  |  |
| Scores >2SDs below average | 14,566 | 14,605 | 14,649 | 14,609 | 14,667 |  |
| Scores ≤2SDs below average | 984 | 945 | 902 | 941 | 883 |  |
| Crude OR | 1.00 | 0.96 (0.87, 1.05) | 0.91 (0.83, 1.00) | 0.95 (0.87, 1.05) | **0.89 (0.81, 0.98)** | **0.03** |
| Adjusted OR ^2^ | 1.00 | 0.95 (0.87, 1.04) | **0.91 (0.83, 1.00)** | 0.95 (0.86, 1.04) | **0.89 (0.81, 0.98)** | **0.04** |
| **Problem Solving** |  |  |  |  |  |  |
| Scores >2SDs below average | 14,621 | 14,727 | 14,728 | 14,742 | 14,765 |  |
| Scores ≤2SDs below average | 929 | 823 | 823 | 808 | 785 |  |
| Crude OR | 1.00 | **0.88 (0.80, 0.97)** | **0.88 (0.80, 0.97)** | **0.86 (0.78, 0.95)** | **0.84 (0.76, 0.92)** | **0.0006** |
| Adjusted OR ^2^ | 1.00 | **0.88 (0.80, 0.97)** | **0.88 (0.80, 0.97)** | **0.87 (0.78, 0.95)** | **0.84 (0.76, 0.92)** | **0.001** |
| **Personal-Social** |  |  |  |  |  |  |
| Scores >2SDs below average | 15,051 | 15,085 | 15,099 | 15,075 | 15,064 |  |
| Scores ≤2SDs below average | 499 | 465 | 452 | 475 | 486 |  |
| Crude OR | 1.00 | 0.93 (0.82, 1.06) | 0.90 (0.79, 1.03) | 0.95 (0.84, 1.08) | 0.97 (0.86, 1.11) | 0.8 |
| Adjusted OR ^2^ | 1.00 | 0.91 (0.80, 1.04) | 0.89 (0.78, 1.01) | 0.94 (0.82, 1.06) | 0.98 (0.86, 1.11) | 0.9 |

^1^ Dietary intake between learning of pregnancy and the second/third trimester. Quintile medians in g/day adjusted energy intake using the residual method.

^2^ Covariates were adjusted for age, previous deliveries, pre-pregnancy BMI, highest maternal educational level, annual household income, marital status, alcohol intake, smoking status, physical activity, employment status, presence of congenital anomaly, child’s sex, and use of EPA and/or DHA supplementation.

Odds ratios and *p* values for trend were calculated by logistic regression analysis. Values in bold indicate significance.

**Supplementary Table 5.** For 6-month-old infants, ORs (95% CIs) of scores at or below -2 SDs of the mean for each development domain according to quintile for n-6/n-3 PUFA intake during pregnancy (n = 81,697).

|  | Quintile for n-6/n-3 PUFA intake | | | | | *p* value |
| --- | --- | --- | --- | --- | --- | --- |
|  | 1 (low) | 2 | 3 | 4 | 5 (high) | for trend |
| **Median intake of n-6/n-3 PUFAs ^1^** | 4.15 | 4.93 | 5.54 | 6.19 | 7.29 |  |
| **Communication** |  |  |  |  |  |  |
| Scores >2SDs below average | 16,023 | 15,990 | 16,023 | 16,023 | 16,027 |  |
| Scores ≤2SDs below average | 316 | 350 | 316 | 317 | 312 |  |
| Crude OR | 1.00 | 1.11 (0.95, 1.29) | 1.00 (0.85, 1.17) | 1.00 (0.86, 1.17) | 0.99 (0.84, 1.16) | 0.5 |
| Adjusted OR ^2^ | 1.00 | 1.12 (0.96, 1.31) | 1.04 (0.88, 1.21) | 1.07 (0.92, 1.26) | 1.09 (0.93, 1.28) | 0.5 |
| **Gross Motor** |  |  |  |  |  |  |
| Scores >2SDs below average | 16,041 | 16,039 | 16,041 | 16,067 | 16,036 |  |
| Scores ≤2SDs below average | 298 | 301 | 298 | 273 | 303 |  |
| Crude OR | 1.00 | 1.01 (0.86, 1.19) | 1.00 (0.85, 1.18) | 0.92 (0.78, 1.08) | 1.02 (0.87, 1.20) | 0.7 |
| Adjusted OR ^2^ | 1.00 | 1.02 (0.87, 1.20) | 1.03 (0.87, 1.21) | 0.96 (0.81, 1.13) | 1.08 (0.92, 1.27) | 0.6 |
| **Fine Motor** |  |  |  |  |  |  |
| Scores >2SDs below average | 15,872 | 15,892 | 15,897 | 15,937 | 15,881 |  |
| Scores ≤2SDs below average | 467 | 448 | 442 | 403 | 458 |  |
| Crude OR | 1.00 | 0.96 (0.84, 1.09) | 0.95 (0.83, 1.08) | **0.86 (0.75, 0.98)** | 0.98 (0.86, 1.12) | 0.3 |
| Adjusted OR ^2^ | 1.00 | 0.97 (0.85, 1.11) | 0.99 (0.86, 1.13) | 0.94 (0.82, 1.07) | 1.10 (0.97, 1.26) | 0.3 |
| **Problem Solving** |  |  |  |  |  |  |
| Scores >2SDs below average | 15,702 | 15,659 | 15,699 | 15,729 | 15,676 |  |
| Scores ≤2SDs below average | 637 | 681 | 640 | 611 | 663 |  |
| Crude OR | 1.00 | 1.07 (0.96, 1.20) | 1.01 (0.90, 1.12) | 0.96 (0.86, 1.07) | 1.04 (0.93, 1.17) | 0.8 |
| Adjusted OR ^2^ | 1.00 | 1.09 (0.97, 1.21) | 1.04 (0.93, 1.17) | 1.02 (0.91, 1.14) | **1.14 (1.02, 1.27)** | 0.12 |
| **Personal-Social** |  |  |  |  |  |  |
| Scores >2SDs below average | 16,095 | 16,086 | 16,137 | 16,120 | 16,121 |  |
| Scores ≤2SDs below average | 244 | 254 | 202 | 220 | 218 |  |
| Crude OR | 1.00 | 1.04 (0.87, 1.24) | **0.83 (0.68, 1.00)** | 0.90 (0.75, 1.08) | 0.89 (0.74, 1.07) | 0.07 |
| Adjusted OR ^2^ | 1.00 | 1.06 (0.89, 1.26) | 0.86 (0.71, 1.04) | 0.97 (0.81, 1.17) | 0.98 (0.82, 1.18) | 0.5 |

^1^ Dietary intake between learning of pregnancy and second/third trimester.

^2^ Covariates were adjusted for age, previous deliveries, pre-pregnancy BMI, highest maternal educational level, annual household income, marital status, alcohol intake, smoking status, physical activity, employment status, presence of congenital anomaly, child’s sex, and use of EPA and/or DHA supplementation.

Odds ratios and *p* values for trend were calculated by logistic regression analysis. Values in bold indicate significance.

**Supplementary Table 6.** For 1-year-old infants, ORs (95% CIs) of scores at or below -2 SDs of the mean for each development domain according to quintile for n-6/n-3 PUFA intake during pregnancy (n = 77,751).

|  | Quintile for n-6/n-3 PUFA intake | | | | | *p* value |
| --- | --- | --- | --- | --- | --- | --- |
|  | 1 (low) | 2 | 3 | 4 | 5 (high) | for trend |
| **Median intake of n-6/n-3 PUFAs^1^** | 4.15 | 4.94 | 5.53 | 6.19 | 7.28 |  |
| **Communication** |  |  |  |  |  |  |
| Scores >2SDs below average | 15,010 | 15,029 | 15,019 | 15,062 | 15,011 |  |
| Scores ≤2SDs below average | 540 | 521 | 532 | 488 | 539 |  |
| Crude OR | 1.00 | 0.96 (0.85, 1.09) | 0.99 (0.87, 1.11) | 0.90 (0.80, 1.02) | 1.00 (0.88, 1.13) | 0.6 |
| Adjusted OR ^2^ | 1.00 | 0.99 (0.87, 1.12) | 1.02 (0.90, 1.15) | 0.95 (0.83, 1.07) | 1.07 (0.95, 1.21) | 0.5 |
| **Gross Motor** |  |  |  |  |  |  |
| Scores >2SDs below average | 14,606 | 14,629 | 14,616 | 14,752 | 14,612 |  |
| Scores ≤2SDs below average | 944 | 921 | 935 | 798 | 938 |  |
| Crude OR | 1.00 | 0.97 (0.89, 1.07) | 0.99 (0.90, 1.09) | **0.84 (0.76, 0.92)** | 0.99 (0.91, 1.09) | 0.14 |
| Adjusted OR ^2^ | 1.00 | 0.99 (0.90, 1.08) | 1.02 (0.93, 1.12) | **0.87 (0.79, 0.96)** | 1.05 (0.96, 1.16) | 0.9 |
| **Fine Motor** |  |  |  |  |  |  |
| Scores >2SDs below average | 14,616 | 14,645 | 14,586 | 14,672 | 14,577 |  |
| Scores ≤2SDs below average | 934 | 905 | 965 | 878 | 973 |  |
| Crude OR | 1.00 | 0.97 (0.88, 1.06) | 1.04 (0.94, 1.14) | 0.94 (0.85, 1.03) | 1.05 (0.95, 1.15) | 0.6 |
| Adjusted OR ^2^ | 1.00 | 0.98 (0.89, 1.07) | 1.06 (0.96, 1.16) | 0.97 (0.88, 1.07) | 1.09 (1.00, 1.20) | 0.103 |
| **Problem Solving** |  |  |  |  |  |  |
| Scores >2SDs below average | 14,727 | 14,744 | 14,710 | 14,746 | 14,656 |  |
| Scores ≤2SDs below average | 823 | 806 | 841 | 804 | 894 |  |
| Crude OR | 1.00 | 0.98 (0.89, 1.08) | 1.02 (0.93, 1.13) | 0.98 (0.88, 1.08) | 1.09 (0.99, 1.20) | 0.12 |
| Adjusted OR ^2^ | 1.00 | 0.99 (0.90, 1.10) | 1.05 (0.95, 1.16) | 1.01 (0.91, 1.11) | **1.13 (1.03, 1.25)** | **0.02** |
| **Personal-Social** |  |  |  |  |  |  |
| Scores >2SDs below average | 15,052 | 15,061 | 15,079 | 15,093 | 15,089 |  |
| Scores ≤2SDs below average | 498 | 489 | 472 | 457 | 461 |  |
| Crude OR | 1.00 | 0.98 (0.86, 1.11) | 0.95 (0.83, 1.08) | 0.92 (0.80, 1.04) | 0.92 (0.81, 1.05) | 0.12 |
| Adjusted OR ^2^ | 1.00 | 0.99 (0.88, 1.13) | 0.97 (0.86, 1.11) | 0.97 (0.85, 1.10) | 1.00 (0.88, 1.14) | 0.8 |

^1^ Dietary intake between learning of pregnancy and second/third trimester.

^2^ Covariates were adjusted for age, previous deliveries, pre-pregnancy BMI, highest maternal educational level, annual household income, marital status, alcohol intake, smoking status, physical activity, employment status, presence of congenital anomaly, child’s sex, and use of EPA and/or DHA supplementation.

Odds ratios and *p* values for trend were calculated by logistic regression analysis. Values in bold indicate significance.
